# Examining behavioural test sensitivity and locomotor proxies of anxiety-like behaviour in zebrafish

**DOI:** 10.1038/s41598-023-29668-9

**Published:** 2023-03-07

**Authors:** Andréa Johnson, Erica Loh, Ryan Verbitsky, Jordan Slessor, Brian C. Franczak, Melike Schalomon, Trevor J. Hamilton

**Affiliations:** 1 Department of Psychology, MacEwan University, 6-329 City Centre Campus, 10700–104 Avenue, Edmonton, AB T5J 4S2 Canada; 2 Department of Statistical Sciences, University of Alberta, Edmonton, AB Canada; 3 Department of Mathematics and Statistics, MacEwan University, 5-107, City Centre Campus, 10700–104 Avenue, Edmonton, AB T5J 4S2 Canada; 4 Neuroscience and Mental Health Institute, University of Alberta, Edmonton, AB Canada

**Keywords:** Drug discovery, Neuroscience

## Abstract

This study assessed the sensitivity of four anxiety-like behaviour paradigms in zebrafish: the novel tank dive test, shoaling test, light/dark test, and the less common shoal with novel object test. A second goal was to measure the extent to which the main effect measures are related to locomotor behaviours to determine whether swimming velocity and freezing (immobility) are indicative of anxiety-like behaviour. Using the well-established anxiolytic, chlordiazepoxide, we found the novel tank dive to be most sensitive followed by the shoaling test. The light/dark test and shoaling plus novel object test were the least sensitive. A principal component analysis and a correlational analysis also showed the locomotor variables, velocity and immobility, did not predict the anxiety-like behaviours across all behaviour tests.

## Introduction

The zebrafish (*Danio rerio*) has become a well-established model organism for testing and developing novel drugs and studying sub-lethal toxicity of ecotoxins and ecotoxicants^1^. Many factors have influenced the popularity of the zebrafish model, including a fully sequenced genome with a 70–80% homology with the human genome, and a central nervous system that shares all major nuclei, neurotransmitters, and receptors^2–5^. Furthermore, zebrafish demonstrate complex behaviours relevant to modeling fear and anxiety-like behaviour^6^, as well as social behaviours, learning and memory, and defensiveness behaviours^5^. With their emerging popularity in laboratory research, it is essential to assess the reliability and validity of commonly used zebrafish tests used to substantiate the behavioural effects of experimental manipulations. With respect to anxiety, a common variable used to assess zebrafish behaviour, test validation can occur with the use of anxiolytic substances that are known to decrease anxiety-like behaviours^7^. Previous studies have also identified several additional locomotor variables presumed to indicate anxiety-like behaviour, such as reduced exploration and freezing (immobility), as well as increased swimming velocity (also measured as distance moved) and erratic movement^8^. Moving forward, it is essential to determine the relative sensitivity of anxiety-like behaviour tests and the relationship between anxiety-like behaviour and locomotion.

Anxiety-like behaviour is a common measurement used in laboratory experiments where anxiety is inferred by behaviours demonstrating specific responses to perceived or anticipated threats. In animals, fear or anxiety is typically exhibited as increased muscle tension, avoidance or escape behaviour, and an increase or surge of autonomic activity^9^. To assess the effects of anxiolytic substances on zebrafish behaviour, previous studies have used several behavioural models adapted from validated rodent paradigms as measures of anxiety-like responses^7,8,10^. Popular behavioural tests used to quantify anxiety-like behaviours in zebrafish include the novel tank dive test^6,8,11–14^, the light/dark test^15,16^, and group shoaling^17–20^, which assess inherent variables of interest specific to each test. More often than not, locomotion is associated with anxiety-like behaviour. The use of locomotion parameters as an index of anxiety in zebrafish can lead to the conclusion that increased swimming velocity and immobility are measures indicative of heightened anxiety^2,3,14,19,21–25^. However, some studies have suggested that the testing conditions and the behavioural test used may be critical factors^16^ as these two locomotor parameters do not always correspond to the main effect on anxiety found in behavioural paradigms. This inconsistency requires further analysis and complex statistical comparison between variables of interest in the anxiety-like behaviour test and locomotion parameters.

**Figure 1:**
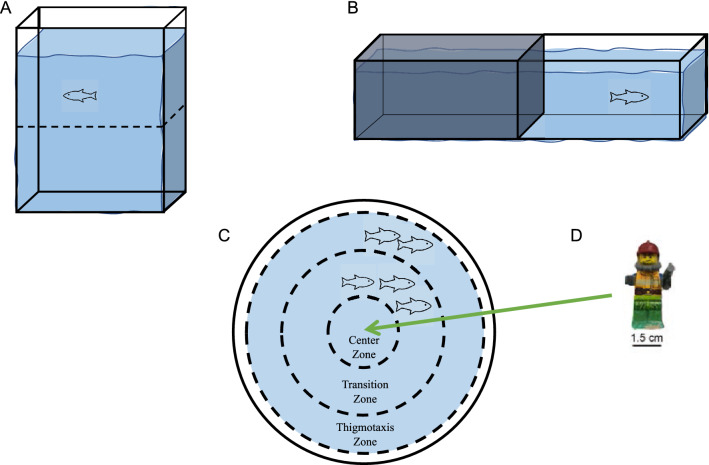
Testing Arenas (**A**) The Novel Tank Dive Test apparatus diagram. The arena measured 5.0 cm wide, 23.3 cm long, 15.0 cm high with a water depth of 13 cm. (**B**) The Light/Dark Test apparatus diagram. The arena measured 94 cm wide, 55.0 cm long, 9.5 cm deep. with a water depth of 5 cm. (**C**) The Shoaling Test apparatus. The arena consisted of a white plastic cylinder (35.0 cm in diameter) filled to a water depth of 5.0 cm. The Shoaling Test and Shoaling plus novel object test zones were generated in EthoVision motion tracking software (centre, transition, and thigmotaxic). (**D**) Object used in the shoaling plus novel object test. Lego figurine with a height of 5 cm.

In this study we investigated the sensitivity of four potential anxiety-like behavioural tests (Fig. 1) with the use of the known anxiolytic drug, chlordiazepoxide^26,27^. We used three concentrations (1, 5, and 15 mg/L) and examined behaviour and locomotion in the novel tank dive test, the light/dark test, shoaling test, and shoaling plus novel object test. Locomotor behaviours, velocity and immobility, were also analyzed. We then performed a principal component analysis (PCA) to compare the anxiolytic effect of chlordiazepoxide on locomotion (velocity and immobility) in relation to the variable of interest in each test. We found the novel tank dive test to be most sensitive, followed by the shoaling test. The light/dark test and shoaling with novel object were demonstrated to be the least sensitive. Furthermore, we found the effect on locomotor variables to be inconsistent with the anxiolytic effect of chlordiazepoxide across anxiety measures.

## Results

### Effects of chlordiazepoxide in the novel tank dive test

#### Tests for significant mean differences across dosage levels

**Figure 2:**
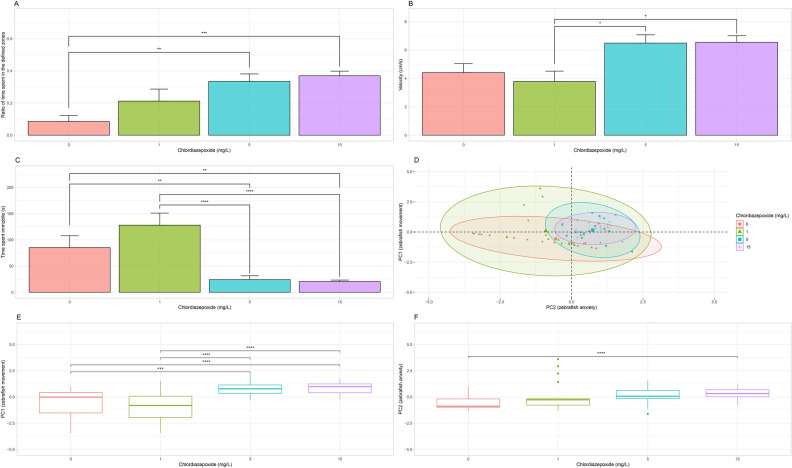
Novel Tank Dive Test. (**A**) Average of the ratio of time fish spent in the defined zones of interest. The ratio of interest increased after treatment with 5 and 15 mg/L chlordiazepoxide. (**B**) Average velocity. Fish move significantly faster after treatment with 5 and 15 mg/L chlordiazepoxide. (**C**) Average immobility. Fish were significantly less immobile after treatment with 5 and 15 mg/L chlordiazepoxide. (**D**) Representation of the ratio of time spent in the defined zones of interest, velocity, and immobility on PC1 and PC2. (**E**) PC1 scores. Significant increases in the PC1 scores were detected at 5 and 15 mg/L of chlordiazepoxide. (**F**) PC2 scores. A significant difference was detected between the 15 mg/L group and the control group. Significant differences are indicated by *(*P* < 0.05), **(*P* < 0.01), ***(*P* < 0.001), and ****(*P* < 0.0001). When applicable, all data is shown as mean ± S.E.M.

Significant differences were detected between the means of every variable of interest when comparing the dosages of chlordiazepoxide in the novel tank dive test. For the ratio of time spent in the defined zones of interest there was a difference between the control group and chlordiazepoxide groups (H(3) = 23.71, *p* < 0.0001). A post-hoc analysis indicated significant increases in the considered ratio at the 5 (0.33 ± 0.05, n = 16, adj. *p* < 0.01) and 15 (0.37 ± 0.03, n = 18, adj. *p* < 0.001) mg/L concentrations compared to the control group (0.08 ± 0.034, n = 17; Fig. 2A). There was a significant main effect on velocity (H(3) = 13.21, *p* < 0.01) with a post-hoc analysis revealing fish in the 5 (6.49 ± 0.59 cm/s, n = 16, adj. *p* < 0.05) and 15 (6.55 ± 0.46 cm/s, n = 18, adj. *p* < 0.05) mg/L groups moved significantly more compared to fish in the 1 mg/L group (3.79 ± 0.72 cm/s, n = 19; Fig. 2B). For immobility, there was also a significant main effect (H(3) = 32.42 and *p* < 0.0001) with a post-hoc analysis finding a significant decrease in immobility in the 5 (24.22 ± 7.45 s, n = 16, adj. *p* < 0.01) and 15 (20.55 ± 2.74 s, n = 18, adj. *p* < 0.01) mg/L groups compared to the control group (85.28 ± 22.70 s, n = 17; Fig. 2D) and a significant decrease in immobility in the 5 (24.22 ± 7.45 s, n = 16, adj. *p* < 0.0001) and 15 (20.55 ± 2.74 s, n = 18, adj. *p* < 0.0001) mg/L groups compared to the 1 mg/L group (128.06 ± 22.99 s, n = 19; Fig. 2C).

#### Assessment of variable relationships

In only the control group, a significant negative correlation was found between velocity and immobility (ρ = -0.87, t(15) = −6.94, *p* ≤ 0.0001). A PCA of the ratio of time spent in the defined zones of interest there across the control and all dosage levels showed that first and second principal components explain 56.49% and 31.44% of the variation in the data, respectively. Velocity (contribution to PC1 = 45.63%) and immobility (contribution to PC1 = 46.66%) contribute very similar amounts of information to the first principal component (PC1), whereas the ratio of time spent in the lower and upper zones (contribution to PC2 = 92.11%) is the strongest contributor of information to PC2. As such, PC1 can be considered a representation of zebrafish movement, whereas PC2 can be considered a representation of zebrafish anxiety-like behaviour (Fig. 2D).

Permutation testing gave evidence that chlordiazepoxide had a significant effect between dosage levels across the values of PC1 (*p* < 0.0001, *aovp*). A post-hoc analysis found a significant difference between the 5 (*p* ≤ 0.001, *perm.test*) and 15 (*p* ≤ 0.0001, *perm.test*) mg/L groups when compared to the control group and between the 5 (p ≤ 0.0001, *perm.test*) and 15 (*p* ≤ 0.0001, *perm.test*) mg/L groups when compared to the 1 mg/L group (Fig. 2E). Further, chlordiazepoxide had a significant effect between dosage levels across the values of PC2 (*p* = 0.05, *aovp*). A post-hoc analysis found a significant difference between the 15 (*p* ≤ 0.0001, *perm.test*) mg/L group when compared to the control group (Fig. 2F).

### Effects of chlordiazepoxide in the light/dark test

#### Tests for significant mean differences across dosage levels

**Figure 3:**
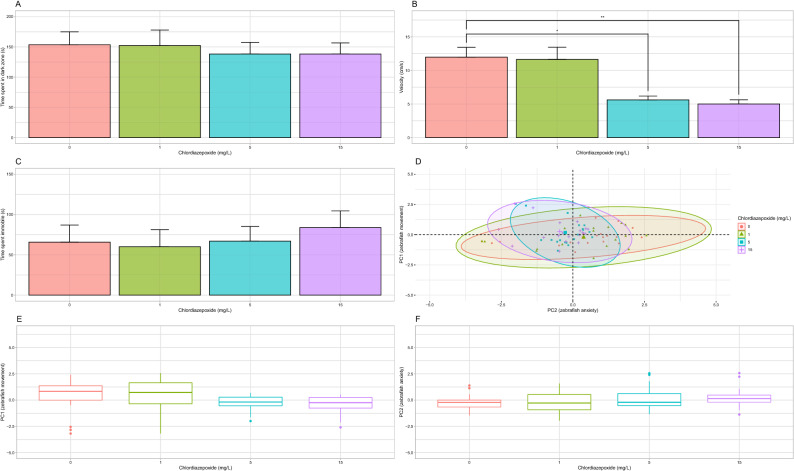
Light/Dark Test. (**A**) Average time spent in dark zone. No significant differences were observed. (**B**) Average velocity. Swimming velocity significantly decreased after treatment with 5 and 15 mg/L chlordiazepoxide. (**C**) Average immobility. No significant differences were observed. (**D**) Representation of time spent in the dark zone, velocity, and immobility on PC1 and PC2. (**E**) PC1 scores. No significant differences were observed. (**F**) PC2 scores. No significant differences were observed. Significant differences are indicated by *(*P* < 0.05) and **(*P* < 0.01). When applicable, all data is shown as mean ± S.E.M.

Chlordiazepoxide did not have a significant effect on time spent in the dark zone (H(3) = 0.38, *p* = 0.94, Fig. 3A). Chlordiazepoxide did have a significant effect on mean velocity between dosages (H(3) = 18.2, *p* < 0.001). A post-hoc analysis indicated significant decreases in velocity between groups treated with 5 (5.61 ± 0.57 cm/s, n = 19, adj. *p* < 0.05) and 15 (5.01 ± 0.62 cm/s, n = 19, adj. *p* < 0.01) mg/L when compared to the control group (11.98 ± 1.46 cm/s, n = 19; Fig. 3B). There was no main effect of chlordiazepoxide on immobility (H(3) = 4.7, *p* = 0.19, Fig. 3C).

#### Assessment of variable relationships

For the control group, significant correlations were found between time spent in the dark zone and velocity (ρ = −0.71, t(17) = −4.10, *p* ≤ 0.001), time spent in the dark zone and immobility (ρ = 0.66, t(17) = 3.64, *p* ≤ 0.01), and between velocity and immobility (ρ = −0.79, t(17) = −5.37, *p* ≤ 0.0001). At the 1 mg/L dosage level, significant correlations were found between time spent in the dark zone and immobility (ρ = 0.62, t(16) = 3.123, *p* ≤ 0.01) and between velocity and immobility (ρ = −0.68, t(17) = −3.74, *p* ≤ 0.01). At the 5 mg/L dosage level, a significant negative correlation was found between velocity and immobility (ρ = −0.80, t(17) = −5.41, *p* ≤ 0.0001). At the 15 mg/L dosage level, a significant negative correlation was found between velocity and immobility (ρ = −0.90, t(17) = −8.29, *p* ≤ 0.0001).

A PCA of the time spent in the dark zone across the control and all dosage levels showed that first and second principal components explain 58.25% and 29.46% of the variation in the data, respectively. Velocity (contribution to PC1 = 43.69%) and immobility (contribution to PC1 = 42.79%) contribute roughly the same amount of information to the first principal component (PC1). Time spent in the dark zone (contribution to PC2 = 86.38%) is the strongest contributor of information to PC2. As such, PC1 can be considered a representation of zebrafish movement, whereas PC2 can be considered a representation of zebrafish anxiety (Fig. 3D). Permutation testing gave no evidence that chlordiazepoxide had a significant effect between dosage levels across the values of PC1 (*p* = 0.25, *aovp, *Fig. 3E) or PC2 (*p* = 0.31, *aovp, *Fig. 3F).

### Effects of Chlordiazepoxide in the Shoaling Test

#### Tests for significant mean differences across dosage levels

**Figure 4:**
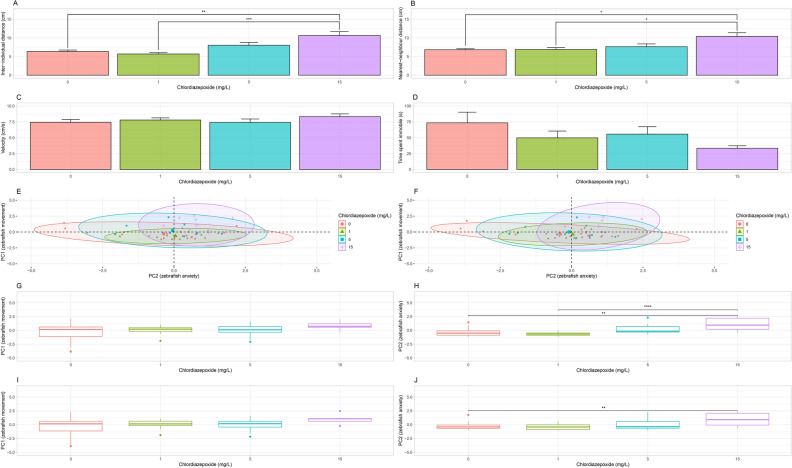
Shoaling Test. (**A**) Average IID. Shoal increased significantly in IID after treatment with 15 mg/L chlordiazepoxide. (**B**) Average NND. Shoal increased significantly in NND after treatment with 15 mg/L chlordiazepoxide. (**C**) Average velocity. No significant differences were observed. (**D**) Average immobility. No significant differences were observed. (**E**) Representation of velocity, immobility, and IID on PC1 and PC2. (**F**) Representation of velocity, immobility, and NND on PC1 and PC2. (**G**) PC1 scores for IID. No significant differences were observed. (**H**) PC2 scores for IID. A significant increase was detected for the 15 mg/L chlordiazepoxide group when compared to the 1 mg/L and control groups. (**I**) PC1 scores on NND. No significant differences were observed. (**J**) PC2 scores on NND. A significant increase was detected for the 15 mg/L the 15 mg/L chlordiazepoxide group when compared to the control group. Significant differences are indicated by *(*P* < 0.05), **(*P* < 0.01), ***(*P* < 0.001), and ****(*P* < 0.0001). When applicable, all data is shown as mean ± S.E.M.

Chlordiazepoxide had a significant effect on inter-individual distance (IID) between groups (H(3) = 18.73, adj. p < 0.001). A post-hoc analysis revealed a significant increase in IID between the control group (6.36 ± 0.39 cm, n = 20, adj. *p* < 0.01) and 1 mg/L group (5.69 ± 0.37 cm, n = 10, adj. *p* < 0.001) when compared to the 15 mg/L group (10.65 ± 1.01 cm, n = 9; Fig. 4A). Chlordiazepoxide also had a significant effect on nearest-neighbour distance (NND) between groups (H(3) = 11.00, *p* < 0.05). A post-hoc analysis showed a significant decrease in NND for the control group (6.8 ± 0.3 cm, n = 20, adj. *p* < 0.05) and 1 mg/L group (6.90 ± 0.51 cm, n = 10, adj. *p* < 0.05) when compared to the 15 mg/L group (10.41 ± 1.00 cm, n = 9; Fig. 4B). However, chlordiazepoxide did not have a significant effect on velocity (F(3, 45) = 0.69, *p* = 0.56, Fig. 4C) or immobility between groups (H(3) = 2.37, *p* = 0.50, Kruskal–Wallis Test, Fig. 4D).

#### Assessment of variable relationships

Significant positive correlations were found between IID and NND for the control group (ρ = 0.84, t(18) = 6.49, *p* ≤ 0.0001), 1 mg/L dosage level (ρ = 0.91, t(8) = 6.12, *p* ≤ 0.001), 5 mg/L dosage level (ρ = 1.00, t(8) = 28.22, *p* ≤ 0.0001), and the 15 mg/L dosage level (ρ = 1.00, t(7) = 33.89, *p* ≤ 0.0001). For the control group, a significant negative correlation was found between velocity and immobility (ρ = −0.80, t(18) = -5.57, *p* ≤ 0.0001). At the 5 mg/L dosage level, a significant negative correlation was found between velocity and immobility (ρ = −0.84, t(5) = −4.38, *p* ≤ 0.0001).

A PCA of IID across the control and all dosage levels showed that first and second principal components explain 59.46% and 32.79% of the variation in the data, respectively. Velocity (contribution to PC1 = 49.28%) and immobility (contribution to PC1 = 48.21%) contribute roughly the same amount of information to the first principal component (PC1). IID (contribution to PC2 = 97.00%) is the strongest contributor of information to PC2. As such, PC1 can be considered a representation of zebrafish movement, whereas PC2 can be considered a representation of zebrafish anxiety (Fig. 4E).

A PCA of NND across the control and all dosage levels showed that first and second principal components explain 62.00% and 30.20% of the variation in the data, respectively. Again, velocity (contribution to PC1 = 45.73%) and immobility (contribution to PC1 = 44.18%) contribute roughly the same amount of information to the first principal component (PC1) and NND (contribution to PC2 = 89.57%) is the strongest contributor of information to PC2. Therefore, once again, we suggest that PC1 be considered a representation of zebrafish movement and PC2 a representation of zebrafish anxiety (Fig. 4F).

Permutation testing gave no evidence that chlordiazepoxide had a significant effect between dosage levels across the values of PC1 for IID (*p* = 0.25, *aovp*, Fig. 4G). However, there is evidence that chlordiazepoxide had a significant effect between dosage levels across the values of PC2 (*p* ≤ 0.0001, *aovp*). A post-hoc analysis found a significant difference between the 15 (*p* ≤ 0.01, *perm.test*) mg/L group and the control group and between the 15 (*p* ≤ 0.0001, *perm.test*) mg/L group and the 1 mg/L group (Fig. 4H).

Permutation testing gave no evidence that chlordiazepoxide had a significant effect between dosage levels across the values of PC1 for NND (*p* = 0.12, *aovp*, Fig. 4I). However, there is evidence that chlordiazepoxide had a significant effect between dosage levels across the values of PC2 (*p* ≤ 0.001, *aovp*). A post-hoc analysis found a significant difference between the 15 (*p* ≤ 0.01, *perm.test*) mg/L group when compared to the control group (Fig. 4J).

### Effects of chlordiazepoxide in the shoaling plus novel object test

#### Tests for significant mean differences across dosage levels

**Figure 5:**
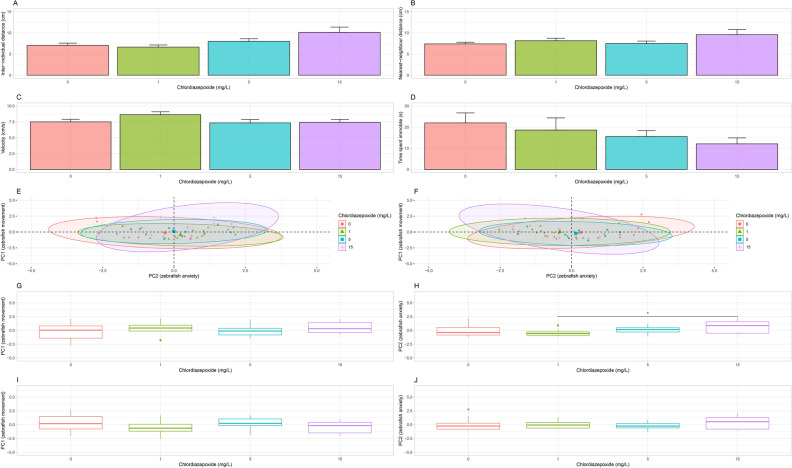
Shoaling Test with Novel Object. (**A**) Average IID. No significant differences were observed. (**B**) Average NND. No significant differences were observed. (**C**) Average velocity. No significant differences were observed. (**D**) Average immobility. No significant differences were observed. All data is shown as mean ± S.E.M. (**E**) Representation of velocity, immobility, and IID on PC1 and PC2. (**F**) Representation of velocity, immobility, and NND on PC1 and PC2. (**G**) PC1 scores for IID. No significant differences were observed. (**H**) PC2 scores for IID. No significant differences were observed. (**I**) PC1 scores on NND. No significant differences were observed. (**J**) PC2 scores on NND. No significant differences were observed. When applicable, all data is shown as mean ± S.E.M.

With the introduction of a novel object, chlordiazepoxide did not have a significant effect between dosage levels for any variable. For IID, a Welch’s ANOVA gave F(3, 19.25) = 2.56 and *p* = 0.09 (Fig. 5A). For NND, a one-way ANOVA gave F(3, 43) = 2.19 and *p* = 0.10 (Fig. 5B). For velocity, a one-way ANOVA gave F(3, 43) = 1.47, *p* = 0.24 (Fig. 5C). For immobility, a Kruskall-Wallis test gave H(3) = 1.26 and *p* = 0.74 (Fig. 5D).

#### Assessment of variable relationships

Significant positive correlations were found between IID and NND for the control group (ρ = 916, t(17) = 9.44, *p* ≤ 0.0001), 1 mg/L dosage level (ρ = 0.97, t(8) = 6.12, *p* ≤ 0.0001), 5 mg/L dosage level (ρ = 1.00, t(8) = 28.82, *p* ≤ 0.0001), and the 15 mg/L dosage level (ρ = 1.00, t(6) = 49.92, *p* ≤ 0.0001). For the control group, a significant negative correlation was found between velocity and immobility (ρ = −0.73, t(17) = −4.46, *p* ≤ 0.001).

A PCA of IID across the control and all dosage levels showed that first and second principal components explain 57.20% and 30.10% of the variation in the data, respectively. Velocity (contribution to PC1 = 45.49%) and immobility (contribution to PC1 = 41.15%) contribute roughly the same amount of information to the first principal component (PC1). IID (contribution to PC2 = 84.38%) is the strongest contributor of information to PC2. As such, PC1 can be considered a representation of zebrafish movement, whereas PC2 can be considered a representation of zebrafish anxiety (Fig. 5E).

A PCA of NND across the control and all dosage levels showed that first and second principal components explain 59.81% and 28.72% of the variation in the data, respectively. Again, velocity (contribution to PC1 = 44.88%) and immobility (contribution to PC1 = 35.68%) contribute roughly the same amount of information to the first principal component (PC1) and NND (contribution to PC2 = 72.26%) is the strongest contributor of information to PC2. Therefore, once again, we suggest that PC1 be considered a representation of zebrafish movement and PC2 a representation of zebrafish anxiety (Fig. 5F).

Permutation testing gave no evidence that chlordiazepoxide had a significant effect between dosage levels across the values of PC1 for IID (*p* = 0.50, *aovp*, Fig. 5G). However, there is evidence that chlordiazepoxide had a significant effect between dosage levels across the values of PC2 for IDD (*p* ≤ 0.05, *aovp*). A post-hoc analysis found a significant difference between the 15 (*p* ≤ 0.05, *perm.test*, Fig. 5H) mg/L group when compared to the 1 mg/L group.

Permutation testing gave no evidence that chlordiazepoxide had a significant effect between dosage levels across the values of PC1 for NND (*p* = 0.46, *aovp*, Fig. 5I) or PC2 for NND (*p* = 0.52, *aovp*, Fig. 5J).

## Discussion

This study investigated the sensitivity of four behavioural paradigms measuring zebrafish anxiety-like behaviour after treatment with the anxiolytic, chlordiazepoxide. Three common tests were used: the novel tank dive test, the light/dark test, and the shoaling test. Additionally, we used a more recently developed paradigm, the shoaling plus novel object test^28^. Chlordiazepoxide significantly decreased anxiety-like behaviour in the novel tank dive test at 5 and 15 mg/L, and in the shoaling test at 15 mg/L. No anxiolytic effects were found in the light/dark test or the shoaling with novel object test. Our results indicate that the novel tank dive test was the most sensitive to anxiolytic effects of chlordiazepoxide followed by the shoaling test. A PCA comparing all variables of interest across all dosage levels and the control group showed that the locomotion and anxiety variables are fully separated in the space of the first two principal components for all tests. Furthermore, for all but the light/dark test, no statistically significant correlations were found between the locomotion and anxiety variables. These results imply that measurements of locomotion and anxiety-like behaviour variables may not be related and locomotion as a proxy for anxiety-like behaviour requires further investigation.

This is not the first study to have found varying sensitivities in behavioural tests in zebrafish^10,29,30^. There are likely many factors that moderate the effects of anxiolytic compounds on behaviour such as the use of habituation tanks, diet, testing environments, fish source, whether the fish were bred in a lab or caught in the wild, or the presence and proximity of experimenters in the testing room, to name a few. Our study eliminated as many factors as possible to study which test would be most sensitive to an anxiolytic, and secondly to compare locomotion parameters to ‘anxiolytic’ parameters within each test.

First, we selected a compound with a circumscribed mechanism of action. While ethanol is often the most commonly used drug in zebrafish research^2,8,20–22,31–40^, it acts on multiple neurotransmitter systems^41^ which complicates the interpretation of behavioural effects. By contrast, the GABA_A_ receptor agonist, chlordiazepoxide, binds to benzodiazepine binding sites on GABA_A_ receptor complexes in the central nervous system resulting in an increased binding of GABA to the GABA_A_ receptor^42^. Chlordiazepoxide was synthesized in the late 1950s and has been used^43,44^ in many organisms such as mice, rats, dogs, monkeys, rabbits^45^, zoo animals^43^, and recently zebrafish^10,22,29,30,46^. In zebrafish, previous studies have found that chlordiazepoxide has sedative effects^29^, reduces locomotor speed^46^, and increases exploratory behaviour^10^. With a baseline of results in other zebrafish studies for us to compare, and a very reliable anxiolytic effect across species (and throughout history), chlordiazepoxide was a clear choice for this study.

### Effects of chlordiazepoxide in the novel tank dive test

The novel tank dive test was the only test to detect anxiolytic effects of chlordiazepoxide at our moderate concentration (5 mg/L) when compared to the control group, which caused a preference for the upper portion of the tank. This behaviour was also observed at 15 mg/L. At these concentrations zebrafish also spent significantly less time in the lower portion of the tank, a behaviour indicating a decreased anxiety response to a novel environment^8^. Our results were not consistent with those of other studies using the novel tank dive test and chlordiazepoxide. Other studies have found that chlordiazepoxide increases bottom-dwelling behaviour at 20 mg/L^29^ and 25 mg/L^10^ in this test; behaviour typically associated with increased anxiety^10,30^. This could be due to methodological differences between studies, such as the strain of fish used or varying dimensions of the experimental testing arena. Each study utilized an entirely novel testing tank to which the fish had never been exposed before experimental testing^10,29^. Our study used a rectangular testing arena. The relatively narrow width of our tank may have evoked a more ecologically valid anxiety-like response. Interestingly, one caveat of the novel tank dive test is that a compound that causes sedation may result in fish spending more time in the bottom section of the tank; a criterion commonly used as a proxy for increased anxiety. In other words, a sedated fish that may in fact be less anxious, may be interpreted as higher in anxiety if the criterion of bottom dwelling is used. This highlights the importance of also validating mobility responses and using other behavioural tests to confirm results.

### Effects of chlordiazepoxide in the shoaling test and the shoaling plus novel object test

The shoaling test was the second most sensitive test and yielded a significant increase in IID and NND with 15 mg/L chlordiazepoxide. These findings indicate an anxiolytic effect of chlordiazepoxide on shoaling behaviour with a decrease in shoal cohesion. Introduction of a novel object eliminated any significant anxiolytic effects of chlordiazepoxide on shoal cohesion measures of IID and NND. The shoaling plus novel object test is not a common test but the paradigm has been employed in previous research. Kwan and colleagues^28^ studied the effects of ocean acidification (OA) on shoaling behaviour in the presence of a novel object in juvenile blacksmith damselfish (*Chromis punctipinnis*), a seawater fish collected from southern California, USA. Despite the differences in fish species (seawater vs. freshwater), a comparison of our studies yielded similar results. Neither the current study nor Kwan et al. ^28^ found significant alterations in IID or NND with the introduction of a novel object between the control and treatment groups.

### Effects of chlordiazepoxide in the light/dark test

Exposure to chlordiazepoxide did not significantly alter the time spent in the light zone relative to the control group. As preference for the light zone is indicative of reduced anxiety in zebrafish^30^, our findings suggest that this method of measuring anxiety was not reduced by chlordiazepoxide at the concentrations used in this study. A similar study found that a low concentration of chlordiazepoxide (0.02 mg/kg) significantly decreased light avoidance, but a high concentration (0.2 mg/kg) did not^30^. Additionally, Sackerman et al. ^10^ found that 25 mg/L of chlordiazepoxide increased the duration of time spent in the light compartment in a modified light/dark test when compared with controls. The anxiolytic effects of chlordiazepoxide on light avoidance are therefore likely to begin between 15 mg/L (the highest concentration in our study) and 25 mg/L^10^ in the light/dark test. The discrepancy in findings may be attributable to differences in apparati used in scototaxis testing procedures. Blaser and Penalosa^47^ found that multiple variables including floor colour, wall colour, and ambient luminosity significantly affected behaviour in the light/dark test. In the present study, the light/dark testing arena employed a white floor with relatively low ambient light, which may have rendered the difference between the light and dark sides of the arena less salient compared to previous research. However, several studies have shown some zebrafish exhibiting an initial preference for the white area of the arena rather than the black area^23,31,47^ which may also account for the absence of a significant preference in our study.

### Effects of chlordiazepoxide on velocity and immobility

Behavioural research has used the speed at which an organism moves (swimming velocity) as a proxy for anxiety. For example, an increase in velocity accompanied by erratic movements has been suggested as indicative of stress and anxiety in fish (Suppl. Table 1). Our study allowed for the comparison of anxiety variables with swimming velocity. If velocity differences are related to changes in anxiety, it would be expected that in each test a significant difference in velocity would appear with the dependent main variable of interest. Analysis of velocity in relation to chlordiazepoxide effects on anxiety-like behaviour yielded inconsistent findings. In the novel tank dive test, chlordiazepoxide had no effect on the swimming velocity of fish in any of the chlordiazepoxide concentrations when compared to the control group, despite significant anxiolytic effects on top dwelling and bottom dwelling at 5 and 15 mg/L. In the shoaling test, chlordiazepoxide had no significant effects on the velocity of the shoal in any of the groups while an anxiolytic effect was observed in shoal cohesion behaviour at 15 mg/L. Surprisingly, in the light/dark test, despite a lack of preference for either zone, a significant decrease in velocity was observed in the 5 and 15 mg/L chlordiazepoxide groups. Overall, changes in velocity due to chlordiazepoxide were not consistent with alterations of the commonly used dependent measures representing anxiety-like behaviour across all tests.

Immobility, the tendency of the organism to remain motionless, is also often used as a proxy for anxiety-like behaviour (Suppl. Table 1). Our analysis of immobility in relation to chlordiazepoxide effects on anxiety-like behaviour also yielded inconsistent results. In the novel tank dive test, immobility did correspond to what we would expect to see given our main effect: in the 5 and 15 mg/L groups chlordiazepoxide reduced bottom dwelling behaviour, increased time in the top zone, and decreased immobility. Here, decreased freezing behaviour would suggest an anxiolytic effect^21^. If velocity were to be a proxy of anxiety-like behaviour, we would expect to see a decrease in swimming speed in relation to a decrease in immobility, however, in this test there were no drug effects on velocity. In the shoaling test we observed a significant increase in shoal size, indicative of anxiolysis, at 15 mg/L but observed no significant alterations in immobility. In the light/dark test, drugs that decrease anxiety usually cause decreased time in the dark zone, yet this was not observed in any treatment group in our study, nor was any change in immobility in this test. However, corresponding to the decrease in velocity that occurred in the 5 and 15 mg/L groups, we would have expected to see a decrease in immobility in these groups as well. In summary, the only test that showed changes in immobility due to chlordiazepoxide was the novel tank dive test. These inconsistencies warrant further study and caution using immobility as a proxy for anxiety-like behaviour.

A correlational analysis conducted on the relationship between the anxiety variables and locomotor behaviour revealed that velocity and immobility are not correlated with time spent in zones or shoal density. A principal component analysis was then used to determine which behaviours most contributed to the responses measured, and whether velocity and immobility contributed to any effects shown on anxiety-like behaviour. The first component (PC1; -axis) can be interpreted as locomotor variables and the second component (PC2; y-axis) can be interpreted as the anxiety level of the fish (see Figs. 2–5). Across all tests, time spent in zones and shoal cohesion contributed to roughly 90% of the variance in behaviour in PC2 on average, and velocity and immobility contributed to roughly 90% of the variation in behaviour in PC1 on average. In the novel tank dive and shoaling test, there were statistically significant differences in dosages across the principal component that represents anxiety. In the novel tank dive, chlordiazepoxide had a significant effect between dosage levels across the values of PC1 and PC2 at 5 and 15 mg/L, and 15 mg/L, respectively, on the ratio of time spent in the defined zones of interest (Fig. 2E,F). In the shoaling test, chlordiazepoxide had a significant effect between dosage levels across the values of PC2 for both IID at 15 mg/L (Fig. 4H and J) when compared to the control group and 1 mg/L group. The PCA results suggest that fish velocity and immobility can be considered a separate effect from the main anxiety variables of interest (time spent in zones and shoal density).

## Conclusion

This study determined that there are significant differences in sensitivity to chlordiazepoxide in anxiety-like behaviour tests, with the novel tank dive test being the most sensitive of the four. In addition to the novel tank dive test, the shoaling test also revealed a dose-dependent effect of chlordiazepoxide. The light/dark test and shoaling plus novel object test yielded no significant changes in anxiety-like behaviour. Notably, this does not mean that these less sensitive tests are less useful; they may be most effective at measuring anxiogenic effects or useful in testing compounds that are more potent. Furthermore, the swimming velocity and tendency to freeze (immobility) were not consistent across tests and results did not parallel those of anxiety variables of interest in all anxiety-like behaviour tests. Therefore, we caution against the use of velocity and immobility as proxies for anxiety-like behaviour as these measures lack validity under our experimental conditions. Taken together, behavioural tests have varying sensitivities and if researchers use one behavioural test and find no effect of a drug or ecotoxicant it does not necessarily mean that there is no behavioural effect outright. A battery of behavioural tests is best to quantify the potential subtle nuances of behavioural changes.

## Methods

### Animals and housing

Adult wildtype zebrafish of mixed gender (*Danio rerio*; n = 411; ~ 50:50, female:male) were obtained from Aquatic Imports (Calgary, AB, Canada). The zebrafish were housed in an Aquatic Habitats (AHAB, Aquatic Ecosystems, Inc., Apopka, FL, USA) three-tier benchtop system in 3L or 10L tanks at a maximum density of 15 or 50 fish, respectively. The habitat water consisted of tap water purified by reverse osmosis and buffered with non-iodized salt, sodium bicarbonate, and acetic acid. Habitat water was continuously re-circulated, UV irradiated, and filtered through 50 µm mechanical and activated carbon chemical filters. The pH and water temperature were monitored daily by an animal care technician. The temperature was maintained between 26 and 30 °C, at a pH of 6.5 to 8.0 and dissolved oxygen levels between 5.0 and 10.0 ppm. Zebrafish were kept on a 12-h light/dark cycle and fed once daily with fish flakes (Gemma Micro 300, Skretting Ltd, France) or shrimp (Omega One Freeze Dried Shrimp Nutri-Treat, Omega Sea Ltd.).

### Experimental procedures

#### Habituation period

Prior to experimental testing with individual behavioural tests, fish were individually transferred by netting to a habituation tank in the testing room. Prior to shoaling tests, the shoal of 5 fish were transferred by netting to a habituation tank in the testing room. Fish habituated in a 3-L polyurethane tank from the housing system for a duration of 25 min. Habituation tanks were fully surrounded by white corrugated plastic walls to reduce the exposure to extraneous visual stimuli. The habituation period was used to acclimate the fish to the ambient light conditions, and to minimize the stress of handling immediately prior to behavioural testing.

#### Drug administration

Following the habituation period, fish were administered 0, 1, 5 or 15 mg/L of chlordiazepoxide (Sigma Aldrich, Ontario, Canada) dissolved in 400 ml of temperature controlled (26–28 °C) reverse osmosis (RO) water. Fish were exposed to the drug solution for a duration of 3 min, based on similar research^48^ and solutions were made fresh each testing day. Control fish underwent the same experimental procedure in a beaker containing 400 ml of pure RO water. All fish used in this study were experimentally and drug naïve. After drug exposure, fish were transferred to the testing arena by netting.

#### Behavioural testing

All behavioural testing was performed during the light phase of the 12 h light/dark cycle in a sound-controlled room with diffuse overhead lighting. Testing was conducted in the mornings prior to feeding. Additionally, control testing was interspersed throughout the treatment conditions to account for any time-of-day effects and other confounding variables. Fish were randomly assigned to either a control group or one of the three treatment groups. During testing, a three-sided enclosure of white corrugated plastic encircled the testing arena to minimize exposure to external stimuli. Water temperature was maintained between 26 °C and 28 °C. The arena was placed on a pad heated to 35 °C to reduce heat loss between trials. Luminance in all testing arenas was measured at ~ 32 cd/m^3^ (cal SPOT photometer; Cooke Corp. CA, USA). A Basler GenICam acA1300-60gc Area Scan video camera (Basler Inc., USA) was suspended approximately 1 m above testing arenas to record zebrafish behaviour. Zebrafish movement was tracked using Noldus EthoVision XT ® tracking software (v. 11.0, Noldus, Wageningen, NL) using differencing settings. Quantification of behaviour began immediately after the fish or shoal was placed in the center of the arena. The time the fish was immobile (immobility) was quantified in EthoVision with a 5% threshold^49,50^. The individual tests, novel tank dive test (n = 80) and light/dark test (n = 81), consisted of ~ 20 fish per group for each 0, 1, 5, and 15 mg/L concentration of chlordiazepoxide. For the shoaling test (n = 250), each shoal consisted of 5 fish. There were 10 trials conducted in each of the shoaling treatment groups, however, in the control group 20 trials were conducted as they were interspersed throughout the testing trials. Data were only excluded from analyses if the tracking software did not acquire data for the total time spent in arena.

#### Novel tank diving test

Zebrafish were released into an elongated arena where their preference for the upper or lower zones of the tank can be calculated by the cumulative time spent in each zone. Decreased time spent in the lower portion of the tank was considered indicative of reduced anxiety. When releasing fish into the testing chamber the net was positioned in the center of the tank, parallel to the longest axis of the arena. The novel tank was 5.0 cm wide by 23.3 cm long and 15 cm high and filled to a water depth of 13 cm. White non-reflective corrugated plastic was affixed to the back of the arena to ensure high contrast between the zebrafish and the background (Fig. 1A). Fish behaviour was tracked for 5-min trials. Behaviours examined for analysis were a ratio of the duration of time spent in defined zones (upper and lower half of the tank) calculated as time spent in the upper zone divided by the sum of the time spent in both the lower and upper zone, average velocity, and time spent immobile.

#### Light/dark test

Zebrafish were released into an arena with one half of the tank surrounded by white corrugated plastic and the other half black corrugated plastic. Preference for either zone was calculated by cumulative time spent in each zone. Decreased time spent in the black zone was considered indicative of decreased anxiety. When the fish were released into the arena, the net was placed facing the long axis of the testing arena at the line dividing the black and white zones, to avoid biasing the fish to either side of the arena. The light/dark arena was 9.4 cm wide by 55 cm long and 9.5 cm deep and was filled to a water depth of 5 cm. The arena walls were surrounded by white and black corrugated plastic placed against the clear Plexiglas walls. White non-reflective corrugated plastic was attached to the bottom of the arena to provide contrast between the fish and the background (Fig. 1B). Each day, the arena was rotated 180 degrees after half of the fish had been tested to eliminate bias resulting from external visual stimuli due to the orientation of the arena. The light/dark test consisted of 5-min trials. Behaviours examined for analysis were duration of time spent in defined zones (light and dark half of the tank), average velocity, and time spent immobile.

#### Shoaling test

Zebrafish were released as a group (shoal) into a circular arena. While the shoal explored the arena, shoal density was calculated by measuring interindividual distance and nearest-neighbour distance. In this test, reduced shoal density was considered indicative of decreased anxiety. The shoaling testing apparatus was a white plastic cylinder (35.0 cm in diameter) filled to a water depth of 5.0 cm. Each shoal consisting of 5 fish was released into the centre of the testing arena. In EthoVision, the arena was segregated equally around the center of the arena into a circular center zone surrounded by annular transition and thigmotaxic zones (Fig. 1C). Behaviour was recorded for 15 min. Shoaling behaviour was analyzed by IID, NND, velocity, and time spent immobile. The average velocity of the shoal was calculated by determining the sum of the mean velocity of each fish and dividing the total by the number of fish in the shoal (5). Similarly, the mean cumulative duration of time spent immobile was calculated for each fish then divided by the total number of fish in the shoal to determine the average immobility of the shoal.

#### Shoaling plus novel object

After 15 min of recording in the shoaling test, a novel object was placed in the center of the arena and fish behaviour was recorded for 5 min. Once again, as the shoal explored the arena with the introduction of a novel object into the center, shoal density was calculated by measuring interindividual and nearest-neighbour distance. Reduced shoal density was considered indicative of decreased anxiety. The novel object was a multicoloured Lego figurine with a height of 5 cm^49–51^ (Fig. 1D). Shoaling behaviour with the introduction of a novel object was analyzed by IID, NND, velocity, and time spent immobile.

### Statistical analysis

The data was analyzed using RStudio Version 2022.12.0 + 353^52^. The analysis is split into two parts. In the first part we tested for differences between the means of the considered variables across the dosage levels. Model assumptions were checked using the Shapiro–Wilk test of normality and the Brown-Forsythe test of variance equality via the R functions *shapiro.test* and *bf.test*. If there was no evidence of non-normality and no evidence of variance inequality, the data were analyzed using a one-way analysis of variance (ANOVA, R function: *aov*). If there was no evidence of non-normality, but there was evidence of variance inequality, the data were analyzed using Welch’s ANOVA (R function: *oneway.test*). If there was evidence of non-normality, the data were analyzed with the Kruskal–Wallis test (R function *kruskal.test*). Post-hoc analyses were performed using either Dunnett’s test, the Games-Howell post hoc test, or Dunn’s test using the R functions *dunnettTest*, *games_howell_test,* or *dunnTest*, respectively. In the second part we studied the relationships between the variables of interest. The goals were to determine whether significant correlations exist and if one, or a combination, of these variables can be considered indicative of anxiety. To study the relationships between the variables we used Pearson’s product moment correlation coefficient via the R function *cor.test*^53^ and a principal component analysis (PCA) via the R function *prcomp*^52^. Following the PCA, we submitted the observations values on the principal components to a permutation testing scheme^54^. The purpose of this permutation testing scheme was to test for statistical differences between each dosage level. We used the R functions *aovp*^55^ and *perm.test*^56^ to run these permutation tests. These functions implement modified versions of the standard one-way ANOVA and multiple comparison procedures used in part one of our analysis. For all permutation tests, we used a seed with value 2022. The 5% significance level was used for assessing statistical significance in all tests. Summary statistics are presented as represented as mean ± S.E.M.

### Ethics statement

All animal care procedures and experiments were approved by the MacEwan University Animal Research Ethics Board (AREB; protocol 05-12-14) and conducted in compliance with the Canadian Council for Animal Care (CCAC) guidelines for the care and use of experimental animals. All methods are reported in accordance with ARRIVE guidelines.

## Supplementary Information

Supplementary Information 1.

Supplementary Information 2.

## Data Availability

Analyzed data from Noldus EthoVision XT **®** tracking software is available in the electronic supplementary material.

## Abbreviations

IID: Interindividual difference
NND: Nearest neighbour distance
