# Supplementary material for: Examining behavioural test sensitivity and locomotor proxies of anxiety-like behaviour in zebrafish
Source: Sci Rep. 2023 Mar 7;13:3768. doi: 10.1038/s41598-023-29668-9 (PMC9992706; doi:10.1038/s41598-023-29668-9)
Supplement: Supplementary file 2 — Supplementary Information 2. [file 41598_2023_29668_MOESM2_ESM.docx]

**Supplementary Table 1**

*Variances of Locomotor Responses in Research with Zebrafish Anxiety-Like Behaviour*

| Study | Test | Treatment | Anxiety-Like Response Measured | Main Effect on Anxiety Interpretation | Locomotor Response Measured | Locomotor Effect on Anxiety  Interpretation^#^ | Consistent Main Effect and Locomotor Effect |
| --- | --- | --- | --- | --- | --- | --- | --- |
| Gerlai et al., 2000 | Novel Tank Dive | ethanol | decreased bottom dwelling | **↓** | decreased immobility | **↓** | Yes |
| Gerlai et al., 2006 | Novel Object Approach | ethanol | decreased avoidance | **↓** | no effect on velocity or immobility | no effect | No |
| Levin et al., 2007 | Novel Tank Dive | nicotine | decreased bottom dwelling | **↓** | decreased velocity | **↓** | Yes |
| Speedie & Gerlai., 2007 | Shoaling | alarm substance | increased shoal cohesion | **↑** | no effect on immobility/increased velocity | no effect /**↑** | No |
| Bencan et al., 2009 | Novel Tank Dive | buspirone | decreased bottom dwelling | **↓** | no effect on velocity | no effect | No |
|  |  | chlordiazepoxide | no effect on behaviour | no effect | decreased velocity | **↓** | No |
|  |  | diazepam | decreased bottom dwelling | **↓** | no effect on velocity | no effect | No |
| Egan et al., 2009 | Novel Tank Dive | caffeine | increased bottom dwelling | **↑** | no effect on immobility/increased velocity | no effect /**↑** | No |
|  |  | fluoxetine | decreased bottom dwelling | **↓** | no effect on immobility/decreased velocity | no effect /**↓** | No |
|  |  | ethanol | decreased bottom dwelling | **↓** | no effect on immobility or velocity | no effect | No |
| Cachat et al., 2010 | Novel Tank Dive | fluoxetine | decreased bottom dwelling | **↓** | no effect on immobility or velocity | no effect | No |
|  |  | ethanol | decreased bottom dwelling | **↓** | decreased immobility/no effect on velocity | **↓**/no effect | No |
|  |  | caffeine | no effect on behaviour | no effect | increased immobility/decreased velocity | **↑**/**↓** | No |
| Wong et al., 2010 | Novel Tank Dive | ethanol | decreased bottom dwelling | **↓** | no effect on immobility or velocity | no effect | No |
|  |  | caffeine | increased bottom dwelling | **↑** | no effect on immobility/increased velocity | no effect/**↑** | No |
|  |  | fluoxetine | decreased bottom dwelling | **↓** | increased immobility/no effect on velocity | **↑**/no effect | No |
| Lau et al., 2011 | Light Dark test | chlordiazepoxide | decreased light avoidance | **↓** | decreased velocity | **↓** | Yes |
|  |  | buspirone | decreased light avoidance | **↓** | no effect on velocity | no effect | No |
| Gebauer et al., 2011 | Shoaling with Novel Tank Dive | clonazepam | decreased shoal cohesion | **↓** | no effect on immobility | no effect | No |
|  |  | bromazepam | decreased shoal cohesion | **↓** | no effect on immobility | no effect | No |
|  |  | diazepam | decreased shoal cohesion | **↓** | no effect on immobility | no effect | No |
|  |  | buspirone | decreased bottom dwelling | **↓** | no effect on immobility | no effect | No |
|  |  | ethanol | decreased shoal cohesion | **↓** | no effect on immobility | no effect | No |
| Grossman et al., 2011 | Novel Tank Dive | piracetam | increased bottom dwelling | **↑** | no effect on immobility | no effect | No |
| Mathur et al., 2011 | Novel Tank Dive | ethanol | decreased bottom dwelling | **↓** | decreased velocity | **↓** | Yes |
|  | Light Dark test | ethanol | decreased light avoidance | **↓** | increased velocity | **↑** | No |
| Maaswinkel et al., 2013 | Novel Tank Dive | buspirone | decreased bottom dwelling | **↓** | no effect on immobility | no effect | No |
|  |  | ethanol | decreased bottom dwelling | **↓** | increased immobility | **↑** | No |
| Maximino et al., 2014 | Light Dark test | buspirone | decreased light avoidance | **↓** | decreased immobility/decreased velocity | **↓** | Yes |
|  |  | caffeine | increased light avoidance | **↑** | increased immobility/increased velocity | **↑** | Yes |
|  |  | diazepam | decreased light avoidance | **↓** | no effect on immobility or velocity | no effect | No |
| Hamilton et al., 2017 | Novel Object Approach | scopolamine | decreased object avoidance | **↓** | no effect on immobility or velocity | no effect | No |
|  |  | ethanol | decreased object avoidance | **↓** | decreased immobility and velocity | **↓** | Yes |
| Johnson et al., 2017 | Novel Object Approach | modafinil | decrease object avoidance | **↓** | no effect on immobility or velocity | no effect | No |
|  |  | ethanol | decreased object avoidance | **↓** | increased immobility/no effect on velocity | **↑**/no effect | No |
| Varga et al., 2018 | Swimming Plus Maze | buspirone | decreased time in deep arms | **↓** | no effect on velocity | no effect | No |
|  |  | chlordiazepoxide | decreased time in deep arms | **↓** | no effect on velocity | no effect | No |
|  |  | caffeine | increased time in deep arms | **↑** | no effect on velocity | no effect | No |
| Zahid et al., 2018 | White Black Test | diazepam | no effect on behaviour | no effect | no effect on immobility/decreased velocity | no effect /**↓** | No |
| Alia et al., 2019 | Novel Tank Dive | caffeine | no effect on behaviour | no effect | no effect on immobility or velocity | no effect | Yes |
|  |  | taurine | no effect on behaviour | no effect | no effect on immobility or velocity | no effect | Yes |
|  | Light Dark test | caffeine | increased light avoidance | **↑** | increased immobility/no effect on velocity | **↑**/no effect | No |
|  |  | taurine | no effect on behaviour | no effect | no effect on immobility or velocity | no effect | Yes |
| Krook et al., 2019 | Novel Object Approach | ethanol withdrawal | increased object avoidance | **↑** | no effect on immobility or velocity | no effect | No |
| Dean et al., 2020 | Novel Object Approach | acute nicotine | decreased object avoidance | **↓** | no effect on immobility or velocity | no effect | No |
|  |  | nicotine withdrawal | no effect on behaviour | no effect | no effect on immobility/decreased velocity | no effect/**↓** | No |
| Hamilton et al., 2021a | Shoaling | benzo[a]pyrene | increased interindividual distance | **↓** | decreased velocity | **↓** | Yes |
|  | Open Field Exploration | benzo[a]pyrene | decreased time in thigmotaxis zone | **↓** | no effect on velocity | no effect | No |
|  | Novel Object Approach | benzo[a]pyrene | decreased object avoidance | **↓** | decreased velocity | **↓** | Yes |
| Hamilton et al., 2021b | Open Field Exploration | CO_2_ | increased time in thigmotaxis zone | **↑** | no effect on velocity or immobility | no effect | No |
|  | Novel Object Approach | CO_2_ | No effect on behaviour | no effect | no effect on velocity/increased immobility | no effect/**↑** | No |
| Szaszkiewicz et al., 2021 | Open Field Test | Limonene | decreased time in thigmotaxis zone | **↓** | increased immobility/decreased velocity | **↑/↓** | No |
|  |  | Myrcene | decreased time in thigmotaxis zone | **↓** | no effect on immobility/decreased velocity | no effect/**↓** | No |
|  |  | Linalool | no effect on behaviour | no effect | no effect on immobility or velocity | no effect | Yes |
|  | Novel Object Approach | Limonene | decreased time in thigmotaxis zone | **↓** | increased immobility/decreased velocity | **↑/↓** | No |
|  |  | Myrcene | decreased time in thigmotaxis zone | **↓** | decreased immobility/no effect on velocity | **↓/**no effect | No |
|  |  | Linalool | no effect on behaviour | no effect | no effect on immobility or velocity | no effect | Yes |
| Abazoid & Gerlai, 2022 | Open Field Exploration | buspirone | decreased time in thigmotaxis zone | **↓** | increased immobility/decreased velocity | **↑**/**↓** | No |

*Note.* Arrows indicate either decreased (↓) or increased (↑) zebrafish anxiety-like behaviour.

#The interpretation based on most literature is that increased swimming velocity indicates increased anxiety and increased immobility indicates increased anxiety.
